# Potential targeted therapeutic approaches in liposarcoma

**DOI:** 10.18632/aging.100945

**Published:** 2016-04-14

**Authors:** Deepika Kanojia, Manoj Garg, H. Phillip Koeffler

**Affiliations:** Cancer Science Institute of Singapore, National University of Singapore, Singapore

**Keywords:** liposarcoma, therapeutics, exome sequencing, tumor heterogeneity, genomic landscape

Liposarcoma (LPS) is rare malignant tumor of fat cells in deep soft tissue that affects adults between the ages of 40 and 60 [1]. This tumor is extremely aggressive with high morbidity and mortality. World Health organization classifies LPS into five subtypes: well-differentiated (WDLPS)/Atypical lipomatous tumors (ALT); dedifferentiated (DDLPS); myxoid (MLPS); pleomorphic (PLPS); and mixed type LPS [2]. Most common modality of treatment is surgical removal of tumor followed by radiation and chemotherapy. The five year survival rate of LPS patients vary from 56%-100% based on different subtypes [3]. Previous genetic studies have focused either on a subset of target genes or one subtype of LPS. Till now no reports have provided a detailed genomic analysis of LPS which included all different subtypes of LPS. Therefore, recently we defined the genomic landscape of LPS using SNP array and whole exome sequencing and identified a spectrum of altered genes and pathways in different subtypes of LPS patients and cell lines [4].

WDLPS and DDLPS subtypes have a characteristic feature of amplified region of chromosome 12q13-15 containing several well-known oncogenes such as MDM2, CDK4 and HMGA2 [5]. SNP array analysis of LPS patients and cell lines identified recurrent amplification of Carboxypeptidase M (CPM) gene only in WDLPS and DDLPS subtypes. We studied in detail CPM gene in vitro and in vivo and showed its involvement in liposarcomagenesis. CPM is a membrane bound enzyme and we found it expressed on the cell surface of LPS cells suggesting it as a potential therapeutic target. Carboxypeptidase activity of CPM is known to regulate the processing of EGF [6]. We found downregulation of CPM expression leads to reduced EGFR signalling and induces apoptosis of LPS cells. Future studies are ongoing detailing further role of enzymatic activity of CPM in EGFR signaling and enhancement of tumor growth. Further, we are developing both a quantitative PCR assay and an ELISA to measure CPM activity in the serum to develop a biomarker to measure minimal residual disease which might aid in monitoring therapy. Specific and selective inhibitors of CPM are not available in the market; therefore, we are also aiming to do screening to find selective and specific small molecules to inhibit CPM enzymatic activity. We proposed CPM as potential therapeutic candidate which could be used as targeted approach to manage CPM amplified WDLPS or DDLPS patients.

Next, whole exome sequencing and targeted exome sequencing identified various known cancer related and novel genes recurrently mutated in different subtypes of LPS. MAPK, ErbB, JAK-STAT, Wnt, apoptosis, cell cycle, DNA replication and repair and axon guidance pathways were found to be potentially involved in liposarcomagenesis. We identified recurrent mutations in previously unidentified genes in LPS associated with DNA damage repair pathways. LPS tumors with DNA repair mutations could be completely dependent on other backup repair pathways for the survival which may be exploited to induce synthetic lethality as therapeutic approach in these tumors.

Interestingly, we reported for the first time multi-region genomic analysis of single LPS patient's tumor signifying intra-tumor mutational and copy number heterogeneity. The current basis for most of the personalized medicine approaches depends on the genomic landscape of a single tumor biopsy sample. Due to the frequent large size of LPS tumors compared to other solid tumors, intra-tumor heterogeneity will lead to difficulties in identifying biomarkers and therapeutic targets. Our preliminary analysis of intra-tumor heterogeneity mandates the need for future detailed studies exploring the evolution of LPS tumors leading to progression.

In summary, our recent work gave insights into global genomic spectrum of LPS cohort for development of novel therapeutic strategies and for understanding the pathogenesis this deadly disease.
